# Identifying key predictors amongst children of under 5 years of age with fever and rashes amidst measles outbreak in Jharkhand: A case control study

**DOI:** 10.1371/journal.pone.0333381

**Published:** 2025-09-29

**Authors:** Dewesh Kumar, Vidya Sagar, Anit Kujur, Sanjay Bhardwaj, Vanesh Mathur, Rahul Kapse, Kaninika Mitra, Rishabh Kumar Rana, Rajan Kumar Barnwal, Bijit Biswas, Syed Irfan Ali, Ravi Ranjan Jha, Tanya Tanu, Ratnesh Sinha, Chandramani Kumar, Abhishek Kumar, Dilip Kumar Paswan, Rahul Chandra, Surendra Sahu, Manish Deo, Atul Kachhap, Santosh Kumar Soren, Priyanka Pawar, Ritika Mukherjee, Divita Sharma, Rajan Kumar, Archisman Mohapatra, Amarendra Kumar

**Affiliations:** 1 Department of Community Medicine, Rajendra Institute of Medical Sciences, Ranchi, India; 2 UNICEF, New York, United States of America; 3 UNICEF, Ranchi, Jharkhand, India; 4 Department of Community Medicine, Shaheed Nirmal Mahto Medical College and Hospital, Dhanbad, India; 5 Department of Community Medicine, Medinirai Medical College and Hospital, Palamu, India; 6 Department of Community and Family Medicine, All India Institute of Medical Sciences, Deoghar, Jharkhand, India; 7 Department of Community Medicine, Manipal Tata Medical College, Manipal Academy of Higher Education, Manipal, India; 8 ESI Hospital, Namkum, Ranchi, Jharkhand, India; 9 Department of Community Medicine, Sheikh Bhikhari Medical College and Hospital, Hazaribagh, India; 10 Narayan Medical College and Hospital, Sasaram, India; 11 Department of Community Medicine, Phulo Jhano Medical College and Hospital, Dumka, India; 12 Generating Research Insights for Development (GRID) Council, Noida, India; 13 Department of Paediatrics, All India Institute of Medical Sciences, Deoghar, Jharkhand, India; 14 WHO, Jharkhand, India; PLOS: Public Library of Science, ETHIOPIA

## Abstract

**Background:**

There has been a recent rise of viral exanthem outbreaks in Jharkhand and other parts of India over the last 2–3 years. This may be due to low immunization coverage of measles vaccination or due to spread of other causes of fever and rash. This study aims to identify the key predictors of fever and rashes among children under five years old by analysing demographic, clinical, and environmental factors.

**Methods:**

This study employed age-matched case-control design to investigate the association between fever with rash (cases) and the absence of such symptoms (controls), along with identifying potential predictors. The study was conducted retrospectively among children aged 0–60 months who presented with fever and rash at various healthcare centres across affected districts in Jharkhand during a measles outbreak from January to May 2023. We included 611 cases and control thus making total sample size to be 1222. Multivariable logistic regression was performed to identify independent risk factors associated with fever and rash during the outbreak.

**Results:**

The mean age for cases was found to be 37.37 ± 22.8 months and while for control it was found to be 25.89 ± 18.6 months. Majority of cases with fever with rash were found among the female child, 50.6% (279) but was not statistically significant. Significant factor as fever and rashes were found to be more amongst the children 200 (68.5%) who had not taken first dose and 325 (53%) of cases who had not taken second dose of measles vaccine with P-value of less than 0.001 and 0.022 respectively. On applying Multivariate analysis religion was 2.5 times more associated and even ethnicity was 3 times more associated with the fever and rash cases.

**Conclusion:**

The study concludes that ethnicity, religion, and caste are important predictors of fever and rash cases. The most important factor is non-receipt of all age-appropriate vaccine as one of key factors for fever and rashes. The sociodemographic factors have a crucial role in transmission of viral exanthem and stakeholders must keep a vigil over these factors while dealing outbreaks of fever with rash disease.

## Introduction

Fever and rashes are perhaps one of the common symptoms in children under five years of age and can be indicative of a wide range of underlying illnesses, from viral infections to more serious systematic diseases requiring prompt intervention [1–3]. Febrile illness is defined as a sudden rise in the body temperature above 38◦ Celsius (100.4 degrees Fahrenheit) without any specific aetiology [4]. Paediatric patients with febrile rash require careful evaluation due to the broad spectrum of differential diagnoses, including conditions like measles, rubella, scarlet fever, and even non-infectious aetiologies [5].

Fever accompanied by a rash contributes significantly to the global disease burden, with a high incidence rate particularly in South and East Asia, sub-Saharan Africa, and the Pacific region [6]. Timely identification and management are crucial to prevent potential complications and ensure appropriate treatment. This mandates a comprehensive approach, including a detailed medical history, thorough physical examination, and appropriate laboratory investigations [6]. Understanding the epidemiology, clinical manifestations, and diagnostic approach to febrile illness with rash in children is essential for healthcare providers to deliver optimal care [7].

Identifying key predictors that differentiate between benign and serious causes of fever with rash is critical for early intervention, minimizing complications, and optimizing healthcare resources [8]. Children under five years of age are particularly vulnerable due to their developing immune systems and heightened susceptibility to infectious diseases. Fever and rashes in this age group may signal a wide range of conditions, from mild viral infections to life-threatening illnesses. Identifying key predictors among these children is crucial for identification of children at higher risk with serious outcomes and prioritize appropriate clinical assessments, especially in resource-limited settings where access to diagnostics and treatment for efficient resource utilization, improved diagnostic accuracy and will guide in clinical decision making [9]. Furthermore, identifying these predictors can help educate caregivers about warning signs, enabling timely medical consultation and empowering them to seek early care. The fever might become life-threatening so prompt diagnosis and early management is very essential [10].

This case-control study aims to identify the key predictors of fever and rashes among children under five years old by analysing demographic, clinical, and environmental factors. This research ultimately seeks to contribute valuable insights to paediatric healthcare and inform targeted strategies for managing febrile illnesses with rash in young children.

## Methodology

### Study design

This study was a case-control design to investigate the association between fever with rashes (cases) and sociodemographic characteristics along with exploring the other potential predictors. We retrospectively performed age matched case-control study among children aged 0–60 months with fever with rashes (cases) who had either a clinical presentation presenting at various health centres in the state during Measles outbreak involving all affected districts between January 2023 and May 2023. First dose of MCV is given at 9 completed months-12 months and second doses of MCV is given at 16–24 months but if missed then can be given 5 years of age under Jharkhand’s routine immunization program. The study has compared the two groups on various factors of interest, such as demographic characteristics, vaccination history, exposure to infected individuals, and healthcare-seeking behaviours. After this outbreak catch up campaign was conducted to cover all the eligible Children of that area.

### Sampling strategy

The selection of cases was involved identifying children diagnosed with fever with rashes during the measles outbreak period in Jharkhand state. These cases were identified through the existing surveillance systems and relevant records. From 16/08/2023–30/08/2023 we retrospectively accessed the data like medical records and investigation reports for research purposes by visiting house to house for all cases and controls. Controls were selected from different adjoining villages and similar age groups as the cases, but without a history of rashes with fever. A systematic random sampling approach was used to select controls from the population.

### Case definitions

We had used the clinical features of measles based on Centre for Disease Control, Atlanta (CDC) criteria for finding the measles cases, which consisted of fever, maculopapular rash, and any symptom between cough, coryza, or conjunctivitis later on confirmed by anti-measles Ig M test among all children with fever and rashes [11].

### Sample size

The required sample size was calculated using a matched case-control formula, with a statistical power of 80%, an assumed exposure proportion among cases of 33.2% (based on WHO reported cases and incidence in India for year 2023), and a case-to-control ratio of 1:1. Without continuity correction, the total calculated minimum sample size was 609 cases and 609 controls. To account for potential exclusions, we enrolled 611 cases and 611 controls, yielding a final analytical sample size of 1,222 participants. Overall total sample size came out to be 1222 in which analysis has been done.

### Data collection

Data collection involved both primary and secondary sources. Primary data were obtained through structured interviews using a pre-tested questionnaire administered to parents or caregivers of the selected cases and controls. The questionnaire collected information on sociodemographic factors, immunization status, contact with suspected or confirmed cases, and healthcare-seeking behaviours. Secondary data, including immunization records and outbreak reports, were retrieved from relevant health institutions and authorities.

### Data analysis

Data was entered in Microsoft Excel and was analysed using Statistical Package for Social Sciences (SPSS) software version 22(SPSS Ic.IBM, NY, US). Descriptive statistics were used to summarize baseline characteristics and vaccination status. Univariate analyses (chi-square tests for categorical variables and t-tests for continuous variables) were conducted to examine differences between cases and controls. Multivariable logistic regression was performed to identify independent risk factors associated with fever and rash during the outbreak. Variables having P value of less than 0.5 in univariate analysis were analysed for Multivariate analysis.Variables gender and ethnicity were treated as potential confounders.

### Ethical considerations

The study was approved by the Institutional Ethics Committee of Rajendra Institute of Medical Sciences (RIMS), Ranchi, under memo number 156 dated 07/08/2023. Written informed consent was obtained from the parents or legal guardians of all participating children after providing complete information regarding the study’s objectives, procedures, risks, and benefits.

## Results

Our present Study was carried out among the 1222 children aged 0–60 months. This Study employing a case control design to investigate the Predictors of fever and rash during the measles outbreak. Out of 1222,611 cases and 611 controls of were taken from the all affected areas of Jharkhand between January 2023 to May 2023.

### Sociodemographic characteristics

Children aged 0–60 months of either gender who had clinical Presentation of fever with rashes were considered as the cases. The male and female group was in a ratio of 1:1.The mean age for cases was found to be 37.37 ± 22.8 months and while for control it was found to be 25.89 ± 18.6 months. Majority of Cases with fever with rash were found among the female Child, 50.6% (279) but was not Statistically Significant. More than half of the cases who were residing in rural areas 62.7% (517) and about one third, 70.3% (284) were following Muslim religion were found to be significant predictors for fever and rash with P-value of less than 0.001. Among cases majority belongs to Scheduled Caste children 60.1% (89) followed by other backward class 56.8% (350) were suffering from fever with rashes and this was found to be statistically Significant with P-value of less than 0.001. Regarding ethnicity non tribals Children 50.2% (487) were affected more than tribals but the factor was not found to be Statistically Significant. Children living in nuclear family 53.9% (371) having fourth birth order 66.2% (49) and were delivered at home 73.8% (138) found to be significant predictors for fever and rash with P value of 0.005 and <0.001 respectively. Fathers Education and occupation was found to be critical as majority of cases were found among those children whose fathers 87.4% (534) had taken education less than 10 years and were daily wages workers 31.9% (195) and both these factors were statistically significant with P-value of less than 0.001.Mothers education less than less than 10 years 88.5% (541) and having housewife by occupation was also found to be key predictors among cases with significant P value of less than 0.001.[Table 1]

**Table 1:** Univariate analysis of fever and rash with their predictors.

Variables		Fever with rash		Total	Test value	P-value
		Control	Case			
**Gender**	**Female**	272(44.5%)	279(45.7%)	551(45.1%)	Fisher’s exact test = 0.730	0.365
	**Male**	339(55.5%)	332(54.3%)	671(54.9%)		
	**Total**	611(100%)	611(100%)	1222(100%)		
**Place of Residence**	**Peri-Urban**	9(1.5%)	0(0.0%)	9(0.8%)	Pearson chi-square = 173.459	**<0.001** ^*^
	**Rural**	307(50.2%)	517(84.7%)	824(67.4%)		
	**Urban**	295(48.3%)	94(15.3%)	389(31.8%)		
	**Total**	611(100%)	611(100%)	1222(100%)		
**Religion**	**Hindu**	428(70%)	217(35.5%)	645(52.8%)	Pearson chi-square = 171.191	**<0.001** ^*^
	**Muslim**	120(19.6%)	284(46.5%)	404(33.1%)		
	**Christian**	32(5.2%)	17(2.8%)	49(4.0%)		
	**Others**	31(5.2%)	93(15.2%)	124(10.1%)		
	**Total**	611(100%)	611(100%)	1222(100%)		
**Caste**	**General**	155(25.4%)	40(6.5%)	195(16%)	Pearson chi-square = 85.873	**<0.001** ^*^
	**OBC**	266(43.5%)	350(57.2%)	616(50.4%)		
	**SC**	59(9.6%)	89(14.6%)	148(12.1%)		
	**ST**	131(21.4%)	132(21.7%)	263(21.5%)		
	**Total**	611(100%)	611(100%)	1222(100%)		
**Ethnicity**	**Non-Tribal**	484(79.2%)	487(50.2%)	971(79.5%)	Pearson chi-square = 1.052	0.591
	**Tribal**	127(20.8%)	124(49.4%)	251(20.5%)		
	**Total**	611(100%)	611(50%)	1222(100%)		
**Type of family**	**Joint**	294(48.1%)	240(45%)	534(43.7%)	Pearson chi-square = 10.509	**0.005** ^*^
	**Nuclear**	317(51.9%)	371(53.9%)	688(56.3%)		
	**Total**	611(100%)	611(50%)	1222(100%)		
**Birth order**	**I**	290(47.5%)	209(42.9%)	499(40.8%)	Pearson chi-square = 45.038	**<0.001** ^*^
	**II**	228(37.3%)	219(49%)	447(36.8%)		
	**III**	64(10.5%)	118(64.5%)	182(14.9%)		
	**IV**	25(4.1%)	49(66.2%)	74(6%)		
	**V**	4(0.6%)	16(80%)	20(1.5%)		
	**Total**	611(100%)	611(100%)	1222(100%)		
**Type of delivery**	**Home**	49(8.0%)	138(22.6%)	187(15.3%)	Fisher’s exact test = 50.011	**<0.001** ^*^
	**Institutional**	562(92%)	473(77.4%)	1035(84.7%)		
	**Total**	611(100%)	611(100%)	1222(100%)		
**Fathers education in years**	**<10 yrs**	277(45.3%)	534(87.4%)	811(66.4%)	Pearson chi-square = 1131.496	**<0.001** ^*^
	**>10 yrs**	334(54.7%)	77(12.6%)	411(33.6%)		
	**Total**	611(50%)	611(50%)	1222(100%)		
**Fathers Occupation**	**Daily wages worker**	116(19%)	195(31.9%)	311(25.4%)	Pearson chi-square = 266.446	**<0.001** ^*^
	**Farming**	118(19.3%)	123(20.1%)	241(19.6%)		
	**Govt. job**	59(9.7%)	10(1.6%)	69(5.6%)		
	**Private job**	191(31.3%)	31(5.1%)	222(18.5%)		
	**Shopkeeper**	73(11.9%)	55(9.0%)	128(10.4%)		
	**Unemployed**	11(1.8%)	7(1.1%)	18(1.5%)		
	**Others**	43(7%)	190(31.1%)	233(19%)		
	**Total**	611(100%)	611(100%)	1222(100%)	Pearson chi-square = 28.544	**<0.001** ^*^
	**Mother’s education in years**	**<10 Yrs**	344(56.3%)	541(88.5%)	885(72.4%)	
		**>10Yrs**	267(43.7%)	70(11.5%)	337(27.6%)	
		**Total**	611(100%)	611(100%)	1222(100%)	
**Mother’s occupation**	**Daily wages worker**	18(2.9%)	9(1.5%)	27(2.2%)	Pearson chi-square = 1049.225	**<0.001** ^*^
	**Housewife**	529(86.6%)	558(91.3%)	1087(89%)		
	**Govt. Job**	16(2.6%)	3(0.5%)	19(1.5%)		
	**Private Job**	20(3.3%)	11(1.8)	31(2.5%)		
	**Part time in own farm**	28(4.6%)	30(4.9)	58(4.8%)		
	**Total**	611(100%)	611(100%)	1222(100%)		

*= Statistically Significant

OBC = Other Backward Class

ST = Scheduled Tribe

SC = Scheduled Caste

Govt. = Government

Among other than Socio-demographic predictors absence of Immunization card 98(84.4%) was found to be Significant factor among the cases and children who had not received all age appropriate vaccines were mostly suffering from fever and rash during the surveillance with P value of less than 0.001.Immunization with first and second dose of Measles vaccine was found to be statistically Significant factor as fever and rashes were found to be more amongst the children 68.5% (200) who had not taken first dose and 53% (325) of cases who had not taken second dose of measles vaccine with P-value of less than 0.001 and 0.022 respectively.[Table 2]

**Table 2:** Univariate analysis of first and second dose of MCV with presence of fever with rash.

Variables		Presence of fever with rash		Total	Test value	P-value
		Controls	Cases			
Immunization card present	Yes	593(97%)	513(84%)	1106(90.5%)	Fischer exact test = 60.959	**<0.001** ^*^
	No	18(3%)	98(16%)	116(9.6%)		
Total		611(100%)	611(100%)	1222(100%)		
Received all age-appropriate vaccines	Yes	533(87.2%)	325(53.2%)	858(70.2%)	Fischer Exact test = 169.281	**<0.001** ^*^
	No	78(12.8%)	286(46.8%)	364(29.8%)		
	Total	611(100%)	611(100%)	1222(100%)		
Received dose of MCV^#^-1	No	94(15.4%)	200(32.7%)	294(24%)	Pearson chi-square = 59.741	**<0.001** ^*^
	Yes	517(84.6%)	411(67.3%)	928(76%)		
Total		611(50%)	611(100%)	1222(100%)		
Received dose of MCV^#^-2	No	289(47.3%)	325(53%)	614(53.2%)	Pearson chi-square = 7.621	**0.022** ^*^
	Yes	322(52.7%)	286(47%)	608(46.8%)		
Total		611(100%)	611(100%)	1222(100%)		

*= Statistically Significant

# MCV = Measles Containing Vaccine

Above table suggest that Child having fever with rashes who had not received first dose of measles containing vaccine were found to be positive for measles when tested for IgM antibody. [Table 3]

**Table 3:** Univariate analysis of first and second dose of MCV with presence of measles among cases(n = 611).

Variables		Result of test		Total	Test value	P-value
		Negative	Only positive for measles			
Child received MCV-1 dose	Before onset of fever and rash	235(98.3%)	4(1.7%)	239(100%)	Pearson chi-square = 7.052	**0.029** ^*^
	After onset of fever and rash	166(96.5%)	6 (3.5%)	172(100%)		
	No	187(93.5%)	13(6.5%)	200(100%)		
	Total	588(96.2%)	23(3.8%)	611(100%)		
Child received MCV-2 dose	Before onset of fever and rash	165(97.6%)	4(2.4%)	169(100%)	Pearson chi-square = 1.723	0.422
	After onset of fever and rash	106(94.6%)	6(5.4%)	112(100%)		
	Not received	317(96.1%)	13(3.9%)	330(100%)		
	Total	588(96.2%)	23(3.8%)	611(100%)		

*= Statistically Significant

In calculating the percentage 411/611 = 67.3% of total 611 cases who were vaccinated with first dose of measles vaccine, therefore yielding a incidence of fever and rashes of 0.67 and a odds of fever and rashes among cases of 0.67/1-.67 = 2.03.For calculating the percentage 281/611 = 46% of total 611 cases who were vaccinated with second dose of measles vaccine, therefore yielding a incidence of fever and rashes of 0.46 and a odds of fever and rashes among cases of 0.46/1-0.46 = 0.85. [Table 4]

**Table 4:** Characteristics of cases with fever with rashes based on exposure of measles vaccine (n = 611).

Measles	Vaccinated	Not vaccinated	Total	Incidence of fever and rashes	Odds of fever and rashes	Pearson chi-square	P value
First dose	411(67.3%)	200(32.7%)	611(100%)	0.67	2.03	56.3088	**.00001** ^*^
Second dose	281(46%)	330(54%)	611(100%)	0.46	0.85		

*= Statistically Significant

On applying Multivariate analysis religion was 2.5 times more associated and even ethnicity was 3 times more associated with the fever and rash cases. Mothers and fathers’ education was 1.4 and 1.6 times more associated among the children who had fever and rashes. Fathers’ education was also 1.13 times more associated with children of fever with rashes. Type of family was 2 times and home delivery were 2.4 times associated as factors for fever with rash. Not Receiving all age-appropriate vaccine was 5.2 times more associated, while after removing confounders first and second dose of Measles was not found to be the predictors for fever and rashes. [Table 5]

**Table 5:** Multivariate analysis of fever and rash with their predictors.

Presence of fever with rash	B	Std. error	Wald	P-value	Odds ratio	95% Confidence interval for exp(B)	
						Lower bound	Upper bound
Gender	0.036	0.204	0.032	0.859	1.037	0.695	1.546
Religion	0.939	0.145	41.857	**0.000** ^*^	2.557	1.924	3.397
Ethnicity	1.130	0.363	9.666	**0.002** ^*^	3.094	1.518	6.307
Caste	0.084	0.101	0.701	0.403	1.088	0.893	1.326
Place of residence	1.394	0.238	34.256	**0.000** ^*^	0.248	0.156	0.396
Mothers’ education in years	0.350	0.050	49.309	**0.000** ^*^	1.418	1.287	1.564
Mothers occupation	0.189	0.116	2.671	0.102	0.828	0.660	1.038
Fathers’ education in years	0.516	0.047	119.699	**0.000** ^*^	1.676	1.528	1.838
Fathers occupation	0.131	0.055	5.618	**0.018** ^*^	1.139	1.023	1.269
Type of family	0.730	0.213	11.730	**0.001** ^*^	2.075	1.366	3.150
Delivery	0.892	0.272	10.784	**0.001** ^*^	2.440	1.433	4.155
Presence of immunization card	0.715	0.407	3.084	0.079	2.043	0.920	4.536
Received all age-appropriate vaccines	1.649	0.255	41.865	**0.000** ^*^	5.202	3.157	8.572
Received one dose of MCV	0.386	0.300	1.658	0.198	1.471	0.817	2.649
Ever received second dose of MCV	0.247	0.248	0.993	0.319	0.781	0.480	1.270

* = Statistically Significant

## Discussion

The current study was undertaken when there were reports of measles outbreaks in different parts of Jharkhand during post COVID pandemic period. Jharkhand was one of the most affected states and has witnessed more than hundreds of outbreaks of fever with rash till 2024 as per Jharkhand State Immunization Office data. From these outbreaks of fever and rash duo, we have had screened and recruited 611 children with fever with rashes along with controls in similar number in 1:1 ratio to identify the key predictors of fever and rash in children. In the areas where outbreaks were reported, the differential diagnoses of this fever with rash cases were made but efforts were made to identify the cause of outbreak which is very crucial for timely public health intervention.

With regards to socio demographic predictors among the children with fever and rashes interesting findings has been observed. Out of 824 children residing in rural areas, 84.7% (517) were suffering from fever with rashes, this finding suggests that the population dwelling in remote rural locations and the hard-to-reach regions are more vulnerable. To avert this situation, we have to strengthen the health services in these areas, as more than 60% of India’s population resides in rural areas [12]. A total of 46.5% (284) cases were Muslim by faith and 57.3% (350) belonged to other backward class which was found to be statistically significant with P value of less than 0.001. Religion is an invisible social determinant that drive public health practice which can affect health of the population particularly [10,13].

More than half of the children suffering from fever and rashes belonged to nuclear family and approximately 25% children’s fathers were working as daily wages workers. And majority of mothers of these children were housewife 89% (1087). the reason might be that Children who grow up with only two parental figures did not get the apt care they need and becomes too isolated if there are financial constrain as compared to joint family which can provide additional help with childcare and finances.

Among predictors other than socio-demographic, absence of immunization card 98 (84.4%) was found to be a significant factor among the cases and children who had not received all age-appropriate vaccines were mostly suffering from fever and rash during the surveillance with P value of less than 0.001. Immunization with Measles vaccine was found to be statistically Significant factor as fever and rashes was found to be more among the children who had not taken second dose of measles vaccine with P-value of 0.022 while it was found that 67.3% (411) of children who had taken first dose had presented with fever and rash with statistically significant P value of 0.001. In this study, cases of measles were detected in immunized children, which suggests that the single dose is not sufficient to confer lasting immunity. However, this finding requires careful interpretation, as vaccination status was obtained from health records or based on parental recall, introducing the potential for bias. Another possible explanation could be the accumulation of unvaccinated individuals within the population, leaving them susceptible to measles but this requires prompt administrative action for enhancing vaccine distribution and coverage, Similar findings was found by Gahr P et al where a measles outbreak occurred due to under vaccination [13].

The current study suggests that child having fever with rashes who had not received first dose of measles containing vaccine were found to be positive for measles when tested for IgM antibody. This predictor was found to be statistically significant with P value of 0.029 similar to findings in the study done by Farhana Rahat et al. [14].

Table 4 suggest that the percentage 411/611 = 67.3% of total 611 cases who were vaccinated with first dose of measles vaccine, therefore yielding a incidence of fever and rashes of 0.67 and a odds of fever and rashes among cases of 0.67/1-0.67 = 2.03 as only one dose is not sufficient to confer lasting immunity, Similar findings was found by Gahr P et al where measles outbreak occurred due to under vaccination [13].For calculating the percentage 281/611 = 46% of total 611 cases who were vaccinated with second dose of measles vaccine, therefore yielding a incidence of fever and rashes of 0.46 and a odds of fever and rashes among cases of 0.46/1-0.46 = 0.85 shows that second dose of measles vaccine had protective effect against children with fever and rashes.

Further evidence from this study was shown in the regression analysis where religion was 2.5 times more associated and even ethnicity was 3 times more associated with the fever and rash cases this might be due to the fact that Jharkhand is a land of difficult terrain and most of the tribals resides in hard to reach area having limited access to healthcare services.Simliar finding was found by systematic review done by Isah Mohammed Bello et al. who also documented limited access to healthcare services as one of predictors for measles outbreak [15].Mothers and fathers’ education was 1.4 and 1.6 times more associated among the children who had fever and rashes, similar findings was found in the study done by Alsofyani BA et al [16], Katrina F. [17] and Brown K et al [18] which showed that parents education is one of most important significant predictors for regarding practices that affect the child’s health.

Fathers’ occupation was also 1.13 times more associated with children of fever with rashes as children belonging to lower socio-economic status are more affected with febrile illness with rashes similar to findings in the study done by Farhana Rahat et al [14]. The nuclear type of family was found to one of the predictors which is 2 times more associated with febrile rashes due to lack of proper care making the children vulnerable to develop fever with rashes. Even in our study home delivery was found as one of the factors which is 2.4 times associated with the cases might be due to the reason of lack of health awareness, as majority of fathers were daily wages worker and mothers were housewife. Not Receiving all age-appropriate vaccine was 5.2 times more associated, while first and second dose of Measles was not found to be the factors for fever and rashes similar to findings in the study done by Farhana Rahat et al [14].

The cases of fever with rash are difficult to diagnose at field level and it always poses a diagnostic challenge in villages and suburban areas [19] . For years, the association of fever with rash has been known, and the differential diagnoses are many. But considering the epidemiology, we can come to a diagnosis and take action in cases of outbreak accordingly. The management depends on the scrupulous history taking and methodological examination, we can come to very near diagnosis and chose the appropriate management [20–21]. But government must make arrangement for diagnostic facilities of febrile rash as in most cases, it results in outbreaks.

## Conclusion

This study fills a conspicuous gap by linking comprehensively and robustly demographics and other predictors with fever and rashes. Place of residence, ethnicity, religion and caste are found to be the demographic predictors. Parents education and occupation also appear to influence the child health. Non-receipt of all age-appropriate vaccine was predicted most strongly as one of key factors for fever and rashes. Predictors identified in this study will guide policy makers, stakeholders and researchers in formulating essential interventions designed with these differential motivations into account. The aim is to avert the future outbreaks, focusing efforts on enhancing measles vaccination coverage, minimizing missed vaccination opportunities and integrating service delivery.

## Supporting information

S1 File Questionnaire. (DOCX)
