# Supplementary material for: Identifying key predictors amongst children of under 5 years of age with fever and rashes amidst measles outbreak in Jharkhand: A case control study
Source: PLoS One. 2025 Sep 29;20(9):e0333381. doi: 10.1371/journal.pone.0333381 (PMC12478947; doi:10.1371/journal.pone.0333381)
Supplement: S1 File — (DOCX) [file pone.0333381.s001.docx]

**Measles RCA _Epidemiological Profile assessment**

1. **Name of State**
2. **Name of District**
3. **Name of Block**
4. **Name of City/Village**
5. **Setting of the Area where child lives**

- Rural
- Urban
- Peri-urban

1. **Name of Monitor**
2. **Date of Visit**
3. **Name of the Child**
4. **Sex of the child**

- Male
- Female
- Any other

1. **Religion**

- Hindi
- Muslim
- Sikh
- Christian
- Others

1. **Caste**
   1. General
   2. OBC
   3. SC
   4. ST
2. **Ethnicity**
   1. **Tribal**
   2. **Non-tribal**
3. **Age of the child in completed years (**If the child is less than 12 completed months Skip to next que)
4. **Age of the child in completed months (**Even if child is more than 12 completed months that is one year please enter completed months)
5. **Birth Order**

- I
- II
- III
- IV
- V

1. **Birth Interval**
   1. **1 Year**
   2. **2 Year**
   3. **More than 3 year**
   4. **NA**
2. **Delivery**
   1. **Delivery**
   2. **Home**
3. **Name of Father**
4. **Father's  education (Completed Years)**
5. **Father's Occupation ***

 Unemployed

 Farming

 Shopkeeper

 Daily wage Worker

 Government Job

 Private Job

 Other

1. **Name of Mother**
2. **Mother's education (Completed Years)**
3. **Mother's Occupation ***

 House Wife

 Daily Wage Worker

 Part timing farming in own field

 Government Job

 Private Job

 Other

1. **Total Family Income (Yearly)**
2. **Type of Family**
   1. **Nuclear**
   2. **Joint**
3. **Who usually accompanies the child to immunization session site? ***

 Mother of the child

 Father of the child

 Grandmother of the child

 Grandfather of the child

 Elder sibling of the child

 Other family members (Aunts or uncles)

 Neighbors or friends

 Community Health worker

 Any Other

1. **Does the child have an immunization card
   ***

 Yes

 No

1. **Has the child received all age appropriate vaccines ***

 Yes

 No

 Maybe

1. **Has the child ever received first dose of MCV?
   ***

 Yes

 No

 Maybe

1. **If Q29 is No what were the reasons?**

 Child still not eligible for Measles first dose

 Did not know when and where to go for Measles Vaccinations

 I visited but there was no vaccine at the immunization site

 Visited the immunization site but health worker did not turn up for immunizing children

 My Child was travelling so missed opportunity to vaccination

 I was scared of the adverse effects of the vaccine

 Vaccine is not for any disease but is to harm my child.

 I don't get any other health service why should I get my child vaccinated?

 I don't trust the government vaccines

 For Immunization services in government health facility l there is long waiting time

 The behavior of health worker is not good and i don't want to take my child for vaccination.

 Could not Vaccinate due to Pandemic restrictions

 We move out of our village during a particular seasons in a year

 I go out for work and usually miss vaccination session as it is during the same time

 Any Other

1. **Has the child ever received second dose of MCV?
   ***

 Yes

 No

 Maybe

1. **If Q31 is No what were the reasons?**

 Child still not eligible for Measles second dose

 Did not know when and where to go for Measles Vaccinations

 I visited but there was no vaccine at the immunization site

 Visited the immunization site but health worker did not turn up for immunizing children

 My Child was travelling so missed opportunity to vaccination

 I was scared of the adverse effects of the vaccine

 Vaccine is not for any disease but is to harm my child.

 I don't get any other health service why should I get my child vaccinated?

 I don't trust the government vaccines

 For Immunization services in government health facility l there is long waiting time

 The behavior of health worker is not good and i don't want to take my child for vaccination.

 Could not vaccinate due to pandemic restrictions

 We move out of our village during a particular season in a year.

 I go out for work and usually miss immunization session as it is conducted during the same time.

 Any Other

1. **Did the child ever suffer from Measles (Fever Rash Surveillance) 
   ***

 Yes

 No

 Maybe

**34. If your child suffered measles infection Did anyone collect blood samples of your child for investigation?
***

 Yes

 No

 Maybe

1. **If blood sampling was done did you receive the report**

 Yes

 No

 Maybe

1. **If you received the report what was the result of test.**

 Only Positive for Measles

 Only Positive for Rubella

 Mixed Measles & Rubella Infection

 Negative

 NA

**NOTE : IF CHILD HAD FEVER AND RASH THEN RESPONSE (Q37 TO Q42)**

1. Did your child received MCV-1 dose
   1. Before onset of fever & rash
   2. After onset of fever & rash
2. Did your child received MCV-2 dose
   1. Before onset of fever & rash
   2. After onset of fever & rash
3. Was there any delayed in receiving MCV-1 dose (age appropriate)
   1. Yes
   2. No
4. If Q39 is yes then how many days? (Response in number of days only)
5. Was there any delayed in receiving MCV-2 dose (age appropriate)
   1. Yes
   2. No
6. If Q41 is yes then how many days? (Response in number of days only)
7. **Do you know the distance of closest immunization site from your house? (In Kms)
   ***
8. **How frequently immunization worker come in your area to vaccinate children
   ***

 Come on a regular basis (Monthly or Quarterly)

 Only when they are called up

 Immunization worker visits but have no plan

 They Visit only during any campaign or special event

 Immunization worker don't visit at all we go to the health facility for vaccination

1. **Are you aware that any measles vaccination campaign happened in your area?  ***

 Yes

 No

 Maybe

1. **Did your child ever received any additional dose of MCV vaccine other than regular two doses.**

 Yes

 No

 Maybe

1. **If you did not receive any additional dose then what were the reasons (Can choose more than one)**

 There was no special campaign for measles vaccination.

 Did not know when and where to go for Measles Vaccinations

 I visited but there was no vaccine at the immunization site

 Visited the immunization site but health worker did not turn up for immunizing children

 My Child was travelling so missed opportunity to vaccination

 I was scared of the adverse effects of the vaccine

 Vaccine is not for any disease but is to harm my child.

 I don't get any other health service why should I get my child vaccinated?

 I don't trust the government vaccines

 For Immunization services in government health facility l there is long waiting time

 The behavior of health worker is not good and i don't want to take my child for vaccination.

 Any Other

 Could not get vaccines due to pandemic restrictions

1. **Did you ever receive any information regarding childhood immunization 
   ***

 Yes

 No

1. **What was the source of information (Can select More than one option)
   ***

 Got information during health facility visit

 Was informed by the Doctor /Nurse

 Health worker informed about immunization

 Through official reminder service (SMS /WhatsApp/Any Other)

 Through Television

 Radio announcement

 Newspaper Advertisements

 Through Social media (Facebook / Instagram), etc

 Got information from family / friends.

 Any Other

**PRACTICE AND BELIEF QUESTION**

1. Do you consider Measles disease as curse from any supernatural/ divine power?

- Yes
- No

2. Do you practice isolation in case of measles due to

- Pathological disease as of scientific knowledge
- Divine curse
- Do not practice isolation.
- Allow children to meet with their parents or restricted group but not everyone.

3. Do you or your family members restrict persons in your family suffering from Measles from taking bath for 9 days?

- Yes
- No

4. Whom do you consult in case of Measles?

- Doctor/ Persons having knowledge of Medical science
- Faith healers
- Both
- None

5. Do you allow children take medicines in case of Measles?

- Yes
- No

6. Do you have any practice of worshipping any divine power in case of Measles disease?

- Yes
- No

7. Do you use neem leaves in case of Fever with rash?

- Yes
- No

8. Any food restriction during active disease?

- Yes
- No
